# Supplementary material for: Pyruvate Dehydrogenase Kinase 1 inhibition mediated oxidative phosphorylation enhancement in cartilage promotes osteoarthritis progression
Source: BMC Musculoskelet Disord. 2023 Jul 20;24:597. doi: 10.1186/s12891-023-06585-6 (PMC10357736; doi:10.1186/s12891-023-06585-6)
Supplement: Supplementary file 1 — Additional file 1. [file 12891_2023_6585_MOESM1_ESM.zip › BP.Gsea.1653623667859/GOBP_AUTOPHAGOSOME_MATURATION.html]

Details for gene set GOBP\_AUTOPHAGOSOME\_MATURATION[GSEA]

|  || Dataset | 55457\_collapsed\_to\_symbols.cls.cls #OA\_versus\_normal.cls.cls #OA\_versus\_normal\_repos |
| Phenotype | cls.cls#OA\_versus\_normal\_repos |
| Upregulated in class | OA |
| GeneSet | GOBP\_AUTOPHAGOSOME\_MATURATION |
| Enrichment Score (ES) | 0.38137895 |
| Normalized Enrichment Score (NES) | 1.456471 |
| Nominal p-value | 0.033980582 |
| FDR q-value | 0.4211145 |
| FWER p-Value | 1.0 |
Table: GSEA Results Summary

  

Fig 1: Enrichment plot: GOBP\_AUTOPHAGOSOME\_MATURATION      
 Profile of the Running ES Score & Positions of GeneSet Members on the Rank Ordered List

  

| SYMBOL | TITLE | RANK IN GENE LIST | RANK METRIC SCORE | RUNNING ES | CORE ENRICHMENT || 1 | CHMP2B | charged multivesicular body protein 2B [Source:HGNC Symbol;Acc:HGNC:24537] | 129 | 0.876 | 0.0495 | Yes |
| 2 | TSG101 | tumor susceptibility 101 [Source:HGNC Symbol;Acc:HGNC:15971] | 284 | 0.763 | 0.0894 | Yes |
| 3 | VTI1B | vesicle transport through interaction with t-SNAREs 1B [Source:HGNC Symbol;Acc:HGNC:17793] | 336 | 0.732 | 0.1352 | Yes |
| 4 | CHMP4A | charged multivesicular body protein 4A [Source:HGNC Symbol;Acc:HGNC:20274] | 526 | 0.649 | 0.1646 | Yes |
| 5 | CHMP5 | charged multivesicular body protein 5 [Source:HGNC Symbol;Acc:HGNC:26942] | 556 | 0.638 | 0.2057 | Yes |
| 6 | ADRB2 | adrenoceptor beta 2 [Source:HGNC Symbol;Acc:HGNC:286] | 596 | 0.625 | 0.2452 | Yes |
| 7 | VAMP8 | vesicle associated membrane protein 8 [Source:HGNC Symbol;Acc:HGNC:12647] | 676 | 0.599 | 0.2797 | Yes |
| 8 | ATG5 | autophagy related 5 [Source:HGNC Symbol;Acc:HGNC:589] | 1142 | 0.488 | 0.2768 | Yes |
| 9 | FYCO1 | FYVE and coiled-coil domain autophagy adaptor 1 [Source:HGNC Symbol;Acc:HGNC:14673] | 1221 | 0.472 | 0.3028 | Yes |
| 10 | CHMP2A | charged multivesicular body protein 2A [Source:HGNC Symbol;Acc:HGNC:30216] | 1269 | 0.464 | 0.3307 | Yes |
| 11 | PIK3R4 | phosphoinositide-3-kinase regulatory subunit 4 [Source:HGNC Symbol;Acc:HGNC:8982] | 1424 | 0.439 | 0.3486 | Yes |
| 12 | WIPI2 | "WD repeat domain, phosphoinositide interacting 2 [Source:HGNC Symbol;Acc:HGNC:32225]" | 1612 | 0.411 | 0.3620 | Yes |
| 13 | VIPAS39 | "VPS33B interacting protein, apical-basolateral polarity regulator, spe-39 homolog [Source:HGNC Symbol;Acc:HGNC:20347]" | 1710 | 0.396 | 0.3814 | Yes |
| 14 | CHMP6 | charged multivesicular body protein 6 [Source:HGNC Symbol;Acc:HGNC:25675] | 2140 | 0.345 | 0.3715 | No |
| 15 | CHMP3 | charged multivesicular body protein 3 [Source:HGNC Symbol;Acc:HGNC:29865] | 2733 | 0.277 | 0.3443 | No |
| 16 | SNAP29 | synaptosome associated protein 29 [Source:HGNC Symbol;Acc:HGNC:11133] | 2885 | 0.262 | 0.3503 | No |
| 17 | LAMP2 | lysosomal associated membrane protein 2 [Source:HGNC Symbol;Acc:HGNC:6501] | 3074 | 0.243 | 0.3522 | No |
| 18 | VAMP7 | vesicle associated membrane protein 7 [Source:HGNC Symbol;Acc:HGNC:11486] | 3380 | 0.214 | 0.3430 | No |
| 19 | VCP | valosin containing protein [Source:HGNC Symbol;Acc:HGNC:12666] | 3518 | 0.201 | 0.3460 | No |
| 20 | MAP1LC3C | microtubule associated protein 1 light chain 3 gamma [Source:HGNC Symbol;Acc:HGNC:13353] | 3943 | 0.161 | 0.3240 | No |
| 21 | VPS33B | VPS33B late endosome and lysosome associated [Source:HGNC Symbol;Acc:HGNC:12712] | 5297 | 0.048 | 0.2220 | No |
| 22 | VPS33A | VPS33A core subunit of CORVET and HOPS complexes [Source:HGNC Symbol;Acc:HGNC:18179] | 7219 | -0.097 | 0.0792 | No |
| 23 | MAP1LC3B | microtubule associated protein 1 light chain 3 beta [Source:HGNC Symbol;Acc:HGNC:13352] | 7323 | -0.104 | 0.0783 | No |
| 24 | CHMP1B | charged multivesicular body protein 1B [Source:HGNC Symbol;Acc:HGNC:24287] | 7339 | -0.105 | 0.0843 | No |
| 25 | CLN3 | "CLN3 lysosomal/endosomal transmembrane protein, battenin [Source:HGNC Symbol;Acc:HGNC:2074]" | 8094 | -0.163 | 0.0367 | No |
| 26 | CALCOCO2 | calcium binding and coiled-coil domain 2 [Source:HGNC Symbol;Acc:HGNC:29912] | 8104 | -0.163 | 0.0471 | No |
| 27 | PIK3C3 | phosphatidylinositol 3-kinase catalytic subunit type 3 [Source:HGNC Symbol;Acc:HGNC:8974] | 8141 | -0.166 | 0.0556 | No |
| 28 | STX17 | syntaxin 17 [Source:HGNC Symbol;Acc:HGNC:11432] | 8466 | -0.191 | 0.0434 | No |
| 29 | UVRAG | UV radiation resistance associated [Source:HGNC Symbol;Acc:HGNC:12640] | 8555 | -0.198 | 0.0500 | No |
| 30 | RUBCNL | rubicon like autophagy enhancer [Source:HGNC Symbol;Acc:HGNC:20420] | 8615 | -0.202 | 0.0592 | No |
| 31 | BECN1 | beclin 1 [Source:HGNC Symbol;Acc:HGNC:1034] | 8644 | -0.204 | 0.0708 | No |
| 32 | ATP2A2 | ATPase sarcoplasmic/endoplasmic reticulum Ca2+ transporting 2 [Source:HGNC Symbol;Acc:HGNC:812] | 9015 | -0.233 | 0.0579 | No |
| 33 | TMEM39A | transmembrane protein 39A [Source:HGNC Symbol;Acc:HGNC:25600] | 9117 | -0.241 | 0.0664 | No |
| 34 | CHMP7 | charged multivesicular body protein 7 [Source:HGNC Symbol;Acc:HGNC:28439] | 9723 | -0.294 | 0.0394 | No |
| 35 | ATG12 | autophagy related 12 [Source:HGNC Symbol;Acc:HGNC:588] | 9776 | -0.299 | 0.0556 | No |
| 36 | CLEC16A | C-type lectin domain containing 16A [Source:HGNC Symbol;Acc:HGNC:29013] | 10013 | -0.323 | 0.0592 | No |
| 37 | CALM1 | calmodulin 1 [Source:HGNC Symbol;Acc:HGNC:1442] | 10136 | -0.334 | 0.0725 | No |
| 38 | VPS16 | VPS16 core subunit of CORVET and HOPS complexes [Source:HGNC Symbol;Acc:HGNC:14584] | 10283 | -0.349 | 0.0848 | No |
| 39 | ATG14 | autophagy related 14 [Source:HGNC Symbol;Acc:HGNC:19962] | 10465 | -0.369 | 0.0959 | No |
| 40 | VMP1 | vacuole membrane protein 1 [Source:HGNC Symbol;Acc:HGNC:29559] | 11018 | -0.432 | 0.0823 | No |
| 41 | RUBCN | rubicon autophagy regulator [Source:HGNC Symbol;Acc:HGNC:28991] | 11162 | -0.452 | 0.1020 | No |
| 42 | CHMP1A | charged multivesicular body protein 1A [Source:HGNC Symbol;Acc:HGNC:8740] | 11401 | -0.487 | 0.1166 | No |
Table: GSEA details [plain text format]

  

Fig 2: GOBP\_AUTOPHAGOSOME\_MATURATION      
 Blue-Pink O' Gram in the Space of the Analyzed GeneSet

  

Fig 3: GOBP\_AUTOPHAGOSOME\_MATURATION: Random ES distribution      
 Gene set null distribution of ES for **GOBP\_AUTOPHAGOSOME\_MATURATION**
